# Supplementary material for: Electrophysiological Properties of Tetraploid Cardiomyocytes Derived from Murine Pluripotent Stem Cells Generated by Fusion of Adult Somatic Cells with Embryonic Stem Cells
Source: Int J Mol Sci. 2023 Mar 31;24(7):6546. doi: 10.3390/ijms24076546 (PMC10095437; doi:10.3390/ijms24076546)
Supplement: Supplementary file 1 [file ijms-24-06546-s001.zip › ijms-1954000-supplementary.pdf]

Supplemental Figures and Tables

Short title: Electrophysiological properties of tetraploid CMs

Index	Page
Figure S1	2
Figure S2	3
Figure S3	4
Figure S4	5
Figure S5	6
Figure S6	7
Figure S7	8
Figure S8	9
Figure S9	10
Figure S10	11
Table S1	13
Table S2	15
Table S3	16

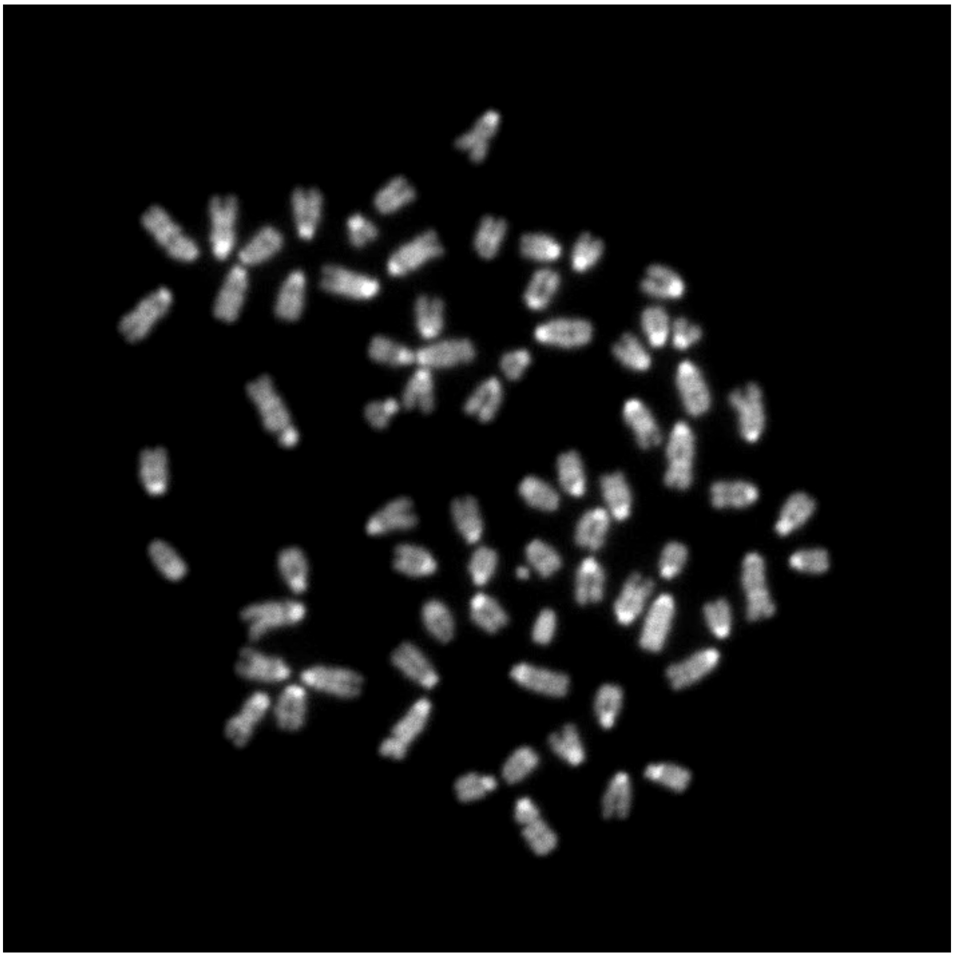

Figure S1. Representative karyotype analysis. Metaphase chromosomal spread prepared from undifferentiated FH-4.2 cells containing 73 chromosomes.

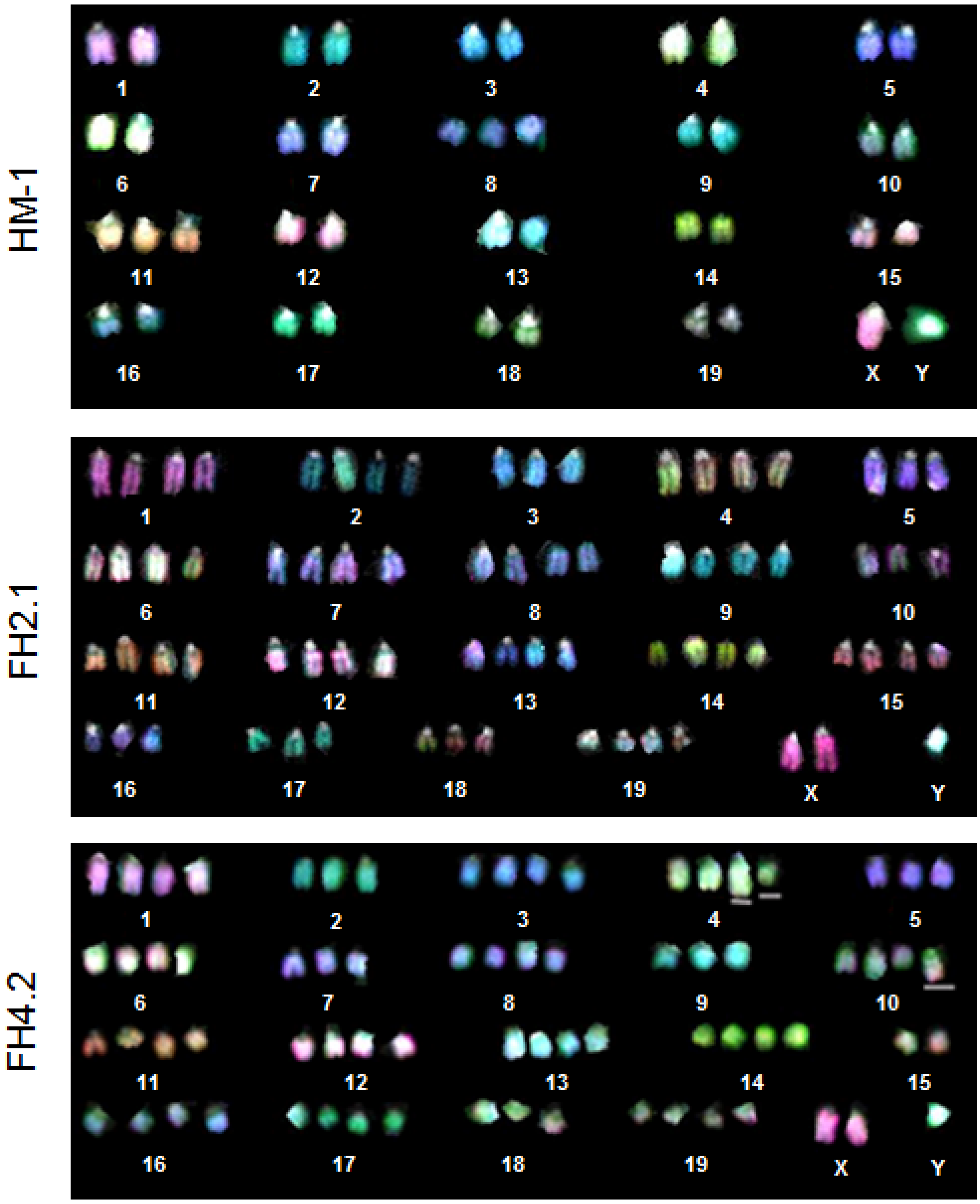

Figure S2. Metaphases of murine HM-1 ES cell and fusion-derived FH-2.1 and FH-4.2 cells analysed by multi colour fluorescence in situ hybridization (mFISH). The metaphase shown for HM-1 ES cells has a diploid set of chromosomes with a gain of a chromosome 8 and chromosome 11 (2n=42, XY, +8, +11). The metaphases shown for FH-2.1 and FH-4.2 cells are pseudotetraploid carrying 73 and 72 chromosomes, respectively. No structural aberrations were detected in chromosomes from FH-2.1 cells. The metaphase shown for FH-4.2 cell has two translocation chromosomes, one consisting of segments of chromosomes 10 and 15 (der10(10;15)) and the other translocation consisting of segments of chromosomes 4 and 14 (der4(4;15)). There is also an enlarged chromosome 4 (dup(4)).

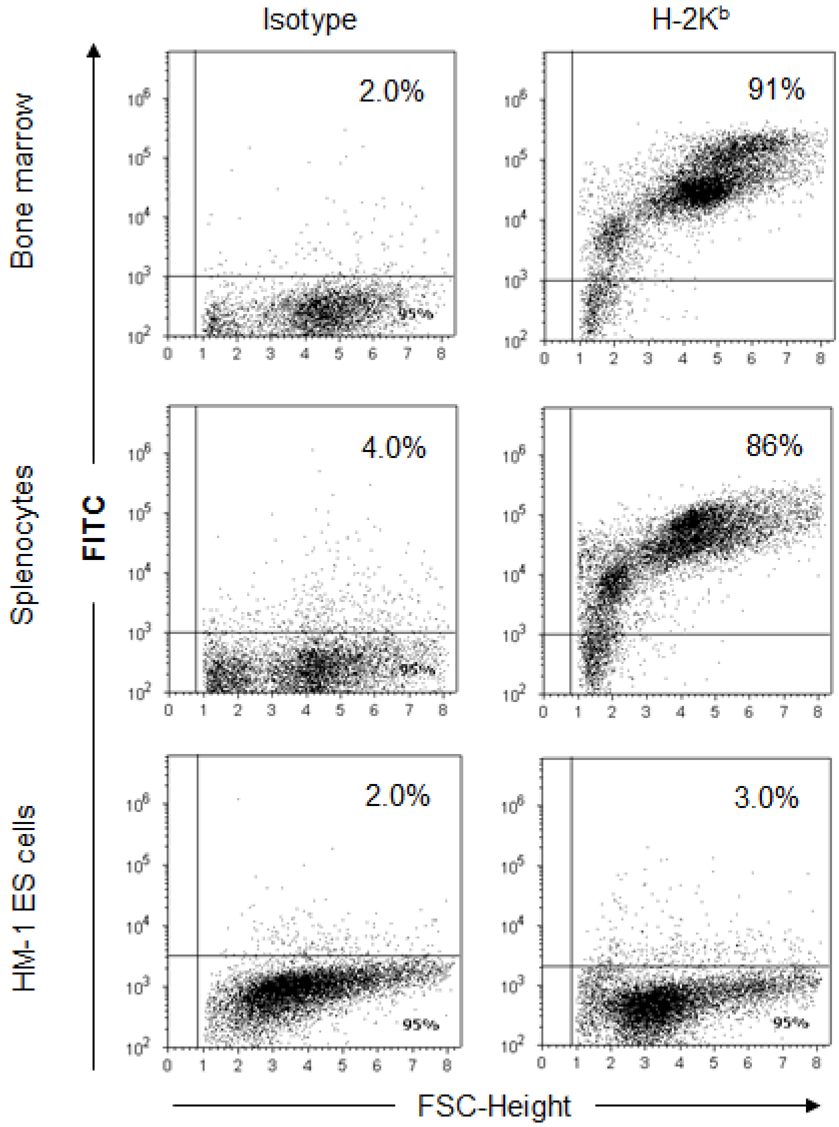

Figure S3. Flow cytometric analysis of major histocompatibility complex class I molecule expression in murine HM-1 ESCs and somatic cells isolated from their mouse strain of origin. Murine adult splenocytes and bone marrow cells were isolated from 129/Ola mouse strain (H-2^b haplotype) and stained with isotype control antibodies or antibodies specific for H-$2K^b$ molecules. Murine HM-1 ESCs (originating from 129/Ola mouse strain) do not express detectable levels of H-$2K^b$ molecules, which is in agreement with our previously published data for CGR8 and D3 ES cell lines (REFERENCES: Abdullah Z, et al. Serpin-6 expression protects embryonic stem cells from lysis by antigen-specific cytotoxic T cells. J Immunol 2007;178:3390-3399, PMID: 17339433, DOI: 10.4049/jimmunol.178.6.3390; Frenzel LP, et al. Lack of NKG2D ligands and ICAM-1 protects murine embryonic stem cell-derived cardiomyocytes from NK cell-mediated lysis. Stem Cells 2009;27:307-316, PMID: 18988711, DOI: 10.1634/stemcells.2008-0528).

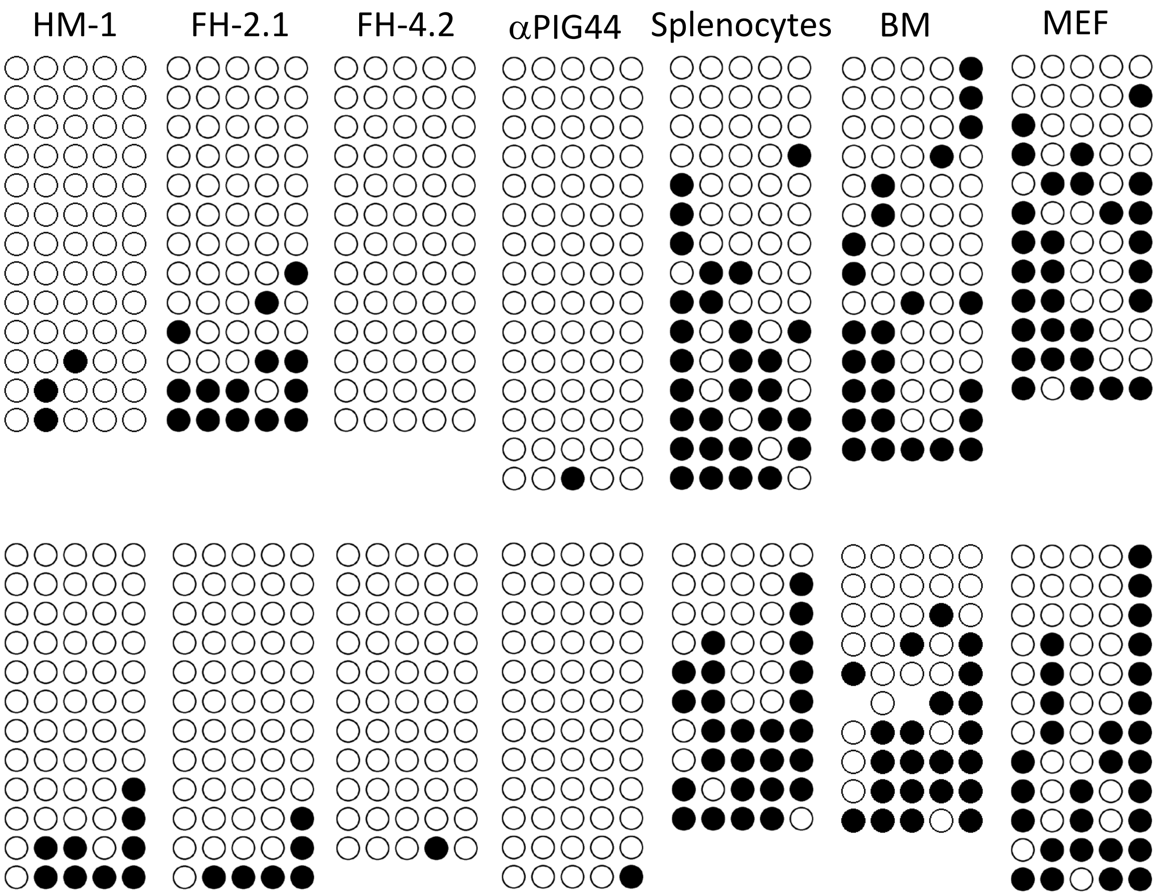

Figure S4. Bisulfite sequencing analysis of *NANOG* and *OCT4 promoter* methylation in indicated pluripotent and somatic cell types**.** DNA methylation pattern of *NANOG* (upper row) and *OCT4* (bottom row) promoter regions are shown for murine HM-1 ES cells, fusion-derived FH-2.1 and FH-4.2 cells, murine ES cells αPIG44 (Kollosov E. et al., J. Exp. Med. 2006), adult murine splenocytes and bone marrow cells (BM), and murine embryonic fibroblasts (MEF). Each line of circles represents a single allele. Filled circles indicate methylated CpGs, empty circles indicate demethylated CpGs.

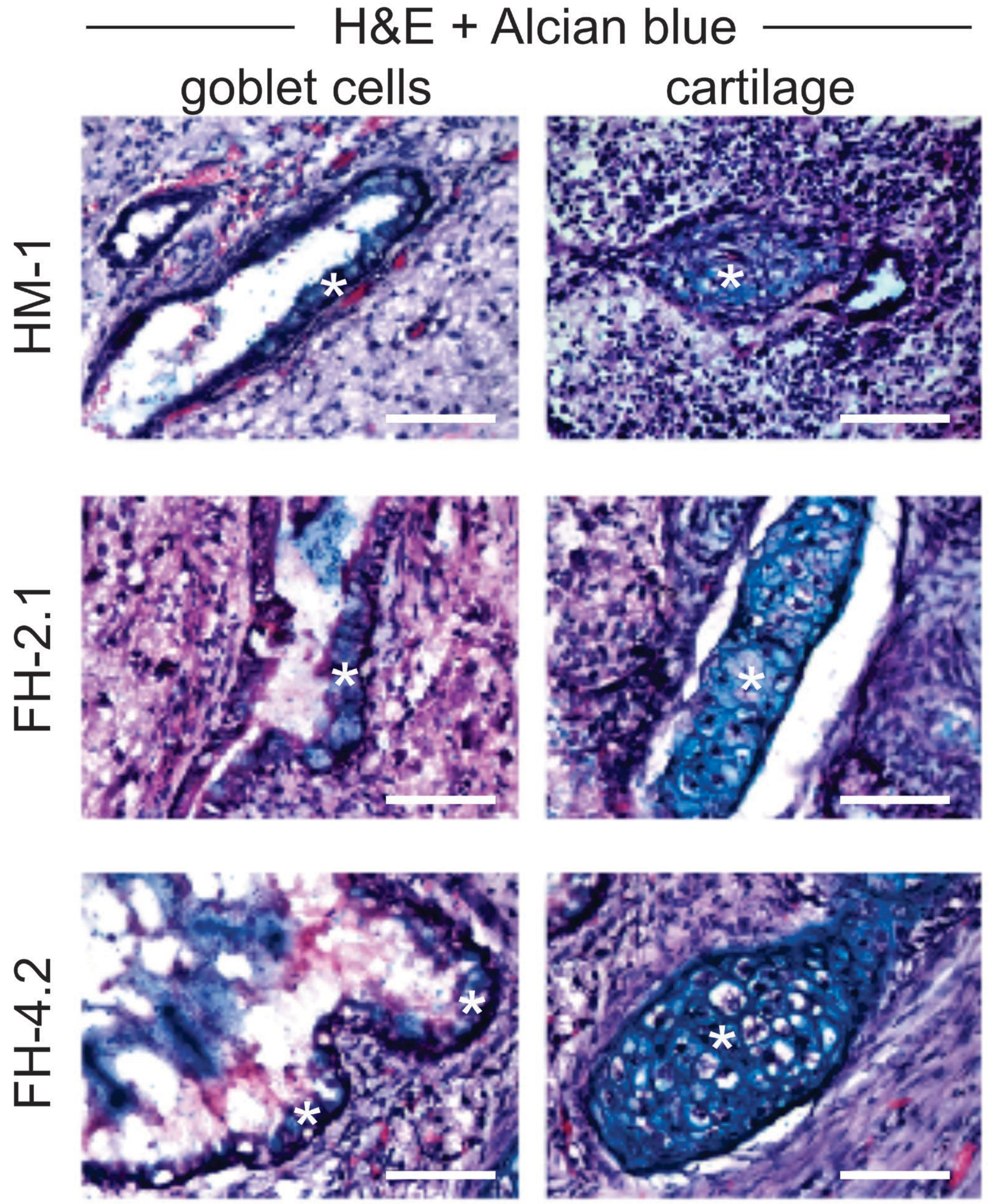

Figure S5. Histological analysis of teratoma derived from murine HM-1 ES cells and fusion-derived FH-2.1 and FH-4.2 ES cell lines. Sections were stained with hematoxylin and eosin (H&E) and Alcian blue to detect acidic polysaccharides in goblet cells (endoderm) and cartilage (mesoderm) as indicated by asterisks. Scale bars: 200 μm.

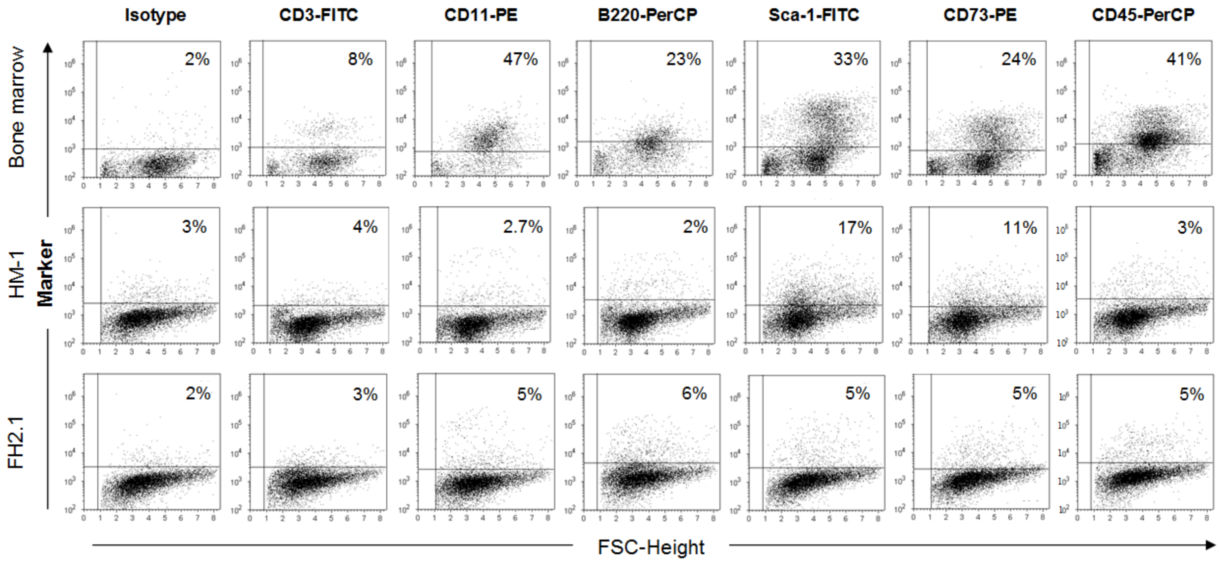

Figure S6. Flow cytometric analysis of somatic cell marker expression in indicated cell types. Bone marrow cells were isolated from wild type DBA/2J mice. All cell types were stained with antibodies specific for indicated lineage-specific cell surface markers. HM-1 ES cells and fusion hybrid FH-2.1 cells did not express significant levels on any of analyzed markers. Numbers in the upper right corner of each graph indicate the percentage of marker-positive cells in this quadrant.

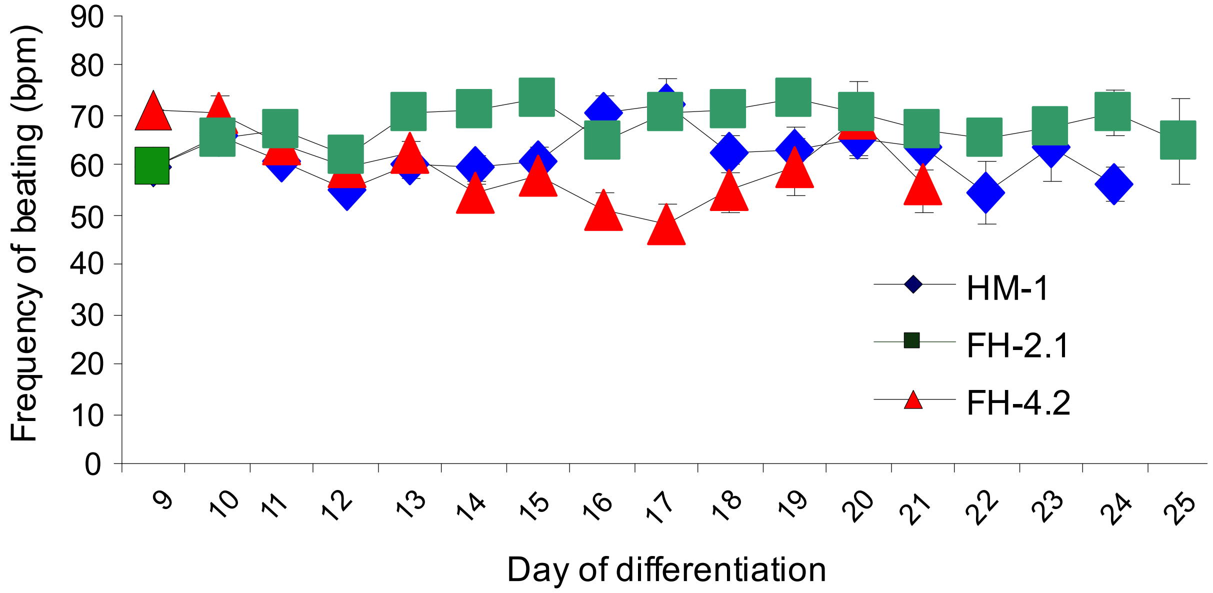

Figure S7. Beating frequencies of spontaneously beating EBs during differentiation. Data are presented as mean ± SEM. bpm – beats per minute.

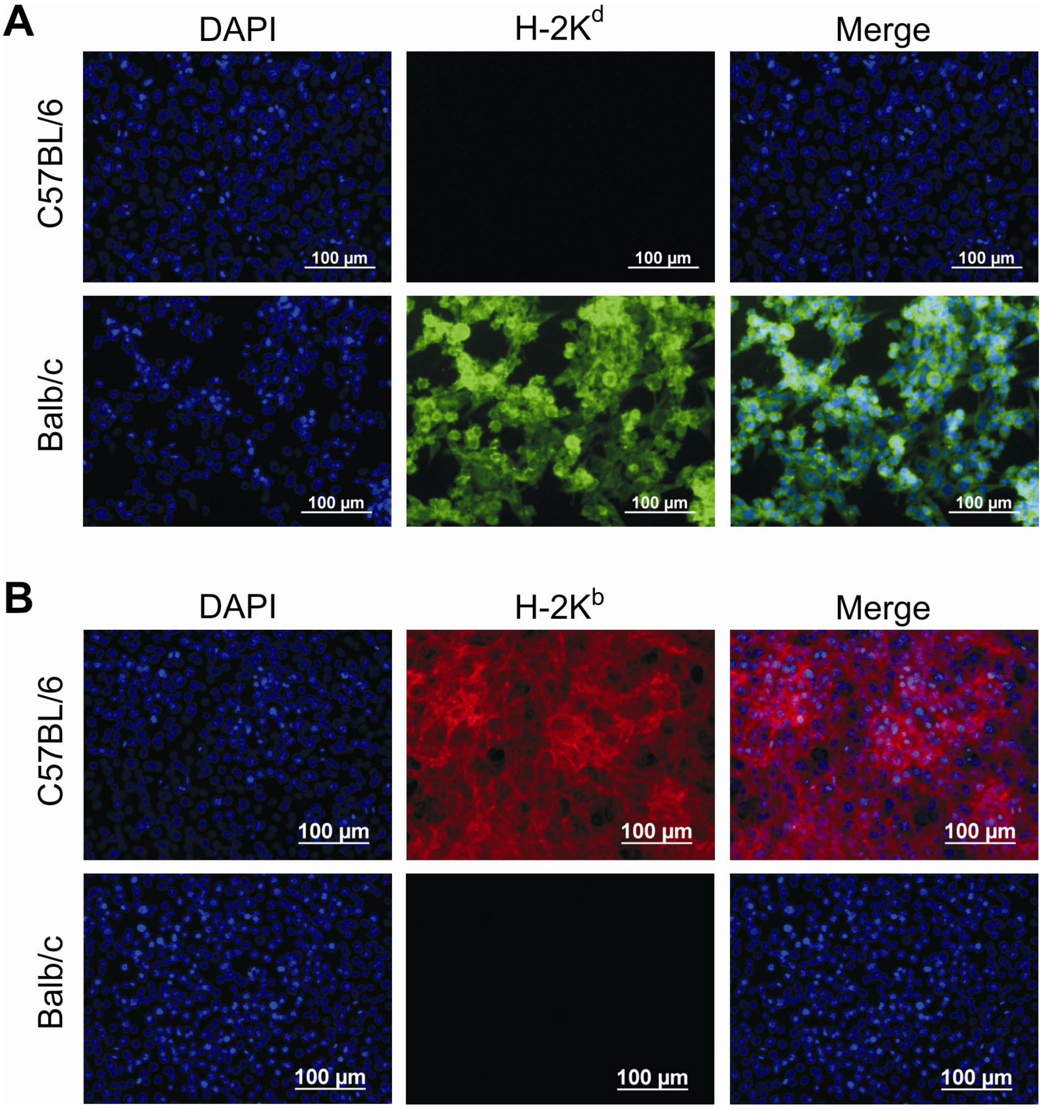

Figure S8. Validation of specificity of antibodies used for detection of H-2K^b and H-2K^d molecules. Immortalized fibroblast cell lines derived from C57BL/6 (H-2K^b hyplotype) and Balb/c (H-2K^d hyplotype) mouse strains were used as positive and negative controls for H-2K^b-H-2K^d-specific antibodies. **(A)** H-2K^d-specific antibodies detected only their respective MHC class I molecules on Balb/c fibroblasts but not on H-2K^b-expressing C57BL/6 fibroblasts. **(B)** In turn, H-2K^b-specific antibodies detected only their respective MHC class I molecules on C57BL/6 fibroblasts but not those on H-2K^d-expressing Balb/c fibroblasts. Detection of H-2K^d (in A) and H-2K^b molecules (in B) was performed by, respectively, AlexaFluor488- (green) and AlexaFluor647-conjugated (red) secondary antibodies against mouse IgG2a. Nuclei were counterstained with DAPI (blue). Scale bars: 100 μm.

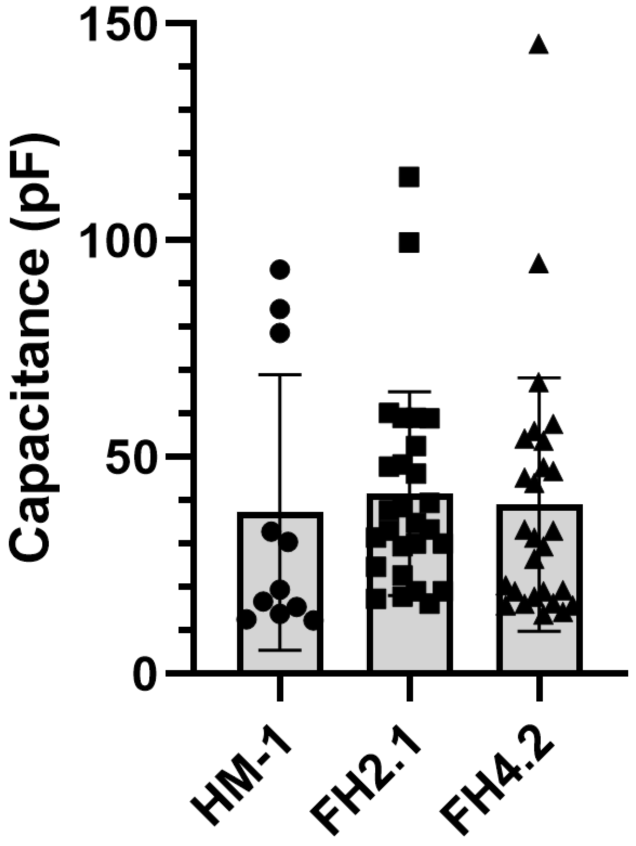

Figure S9. Membrane capacitance of cardiomyocytes derived from diploid murine HM-1 ES cells and near-tetraploid fusion-derived FH-2.1 and FH-4.2 ES cells. Cardiac differentiation of diploid and fusion-derived murine ES cell lines was performed using the hanging drop method. Cardiac clusters were enzymatically dissociated on day 10 of differentiation, plated on fibronectin-coated dishes and single cardiomyocytes used for membrane capacitance analysis as described in the Materials and Methods section. The data are given as mean ± SD and the individual data points in each group are represented by the corresponding symbols. N=11, 27 and 27 for HM1, FH2.1 and FH4.2 cardiomyocytes, respectively. The difference in membrane capacitance between the groups was not statistically significant as determined by the one-way ANOVA ($P>0.05$).

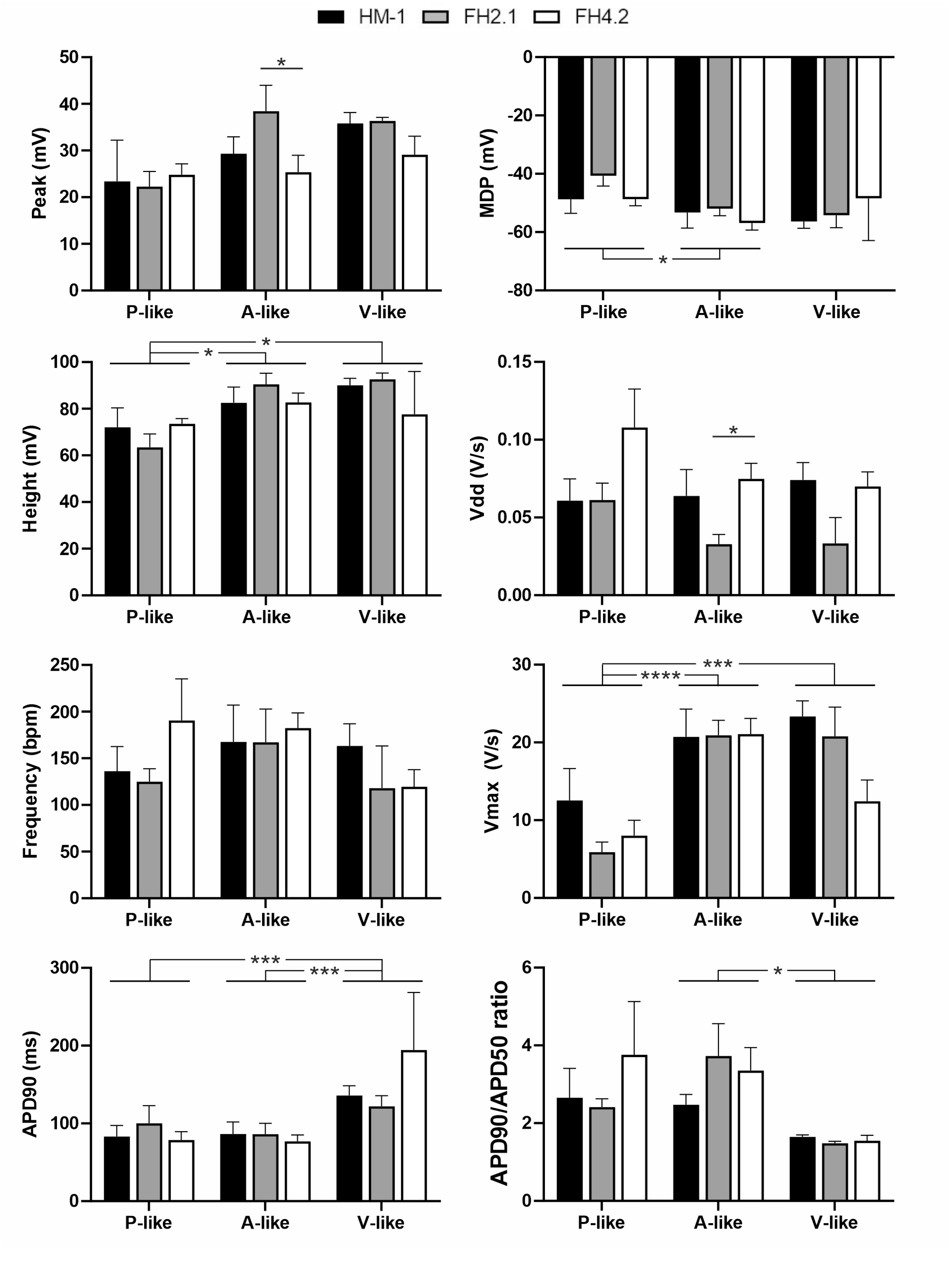

Figure S10. Comparison of action potential (AP) parameters for different subtypes of CMs derived from diploid murine HM-1 ESCs and near-tetraploid fusion-derived hybrid PSCs FH-2.1 and FH-4.2. Spontaneous APs were recorded at day 16-19 of differentiation. Cardiomyocytes were categorized according to their AP morphology to pacemaker-like (P-like), ventricular-like (V-like) and atrial-like (A-like) cells. Statistical analysis of differences between cell lines and between CM-subtypes was performed by two-way ANOVA followed by the post-

hoc Tukey's test to correct for multiple comparisons. Except the significant difference in Peak and Vdd parameters for atrial like cells between FH-2.1 and FH-4.2 CMs, no other statistically significant differences were found in AP parameters between diploid and near-tetraploid CMs within each CM-subtype. Most statistically significant differences were found between different CM-subtypes for MDP (P-like vs A-like), AP height (P-like vs A-like and P-like vs V-like), Vmax (P-like vs A-like and P-like vs V-like), APD90 (P-like vs V-like and A-like vs- V-like) and APD90/APD50 ratio (A-like vs V-like). Data are shown as mean ± SEM. P-values are only given for comparisons with statistically significant differences. *P < 0.05, ***P < 0.001, ****P < 0.0001. Abbreviations: MDP - maximal diastolic potential, Vdd - velocity of diastolic depolarization, Vmax - maximum velocity of depolarization, APD90 - AP duration at 90% repolarization, APD50 - AP duration at 50% repolarization.

Table S1. The list of the reference variant alleles of the single nucleotide polymorphism (SNPs) arbitrarily selected for each chromosome and of PCR primers used for PCR amplification of the corresponding genomic regions and DNA-sequencing to detect polymorphic nucleotides (see Table S2).

Chromosome	Ref. seq.	Primer	Product size	Annealing temp., °C
1	rs3722007	**F:** GGCTTCTGGCTCTGTTTTTG<br>**R:** GGAAACAGCCAATCTTCAGG	483	58
2	rs13476485	**F:** AGGCAAGGTGCTTGTGATCT<br>**R:** TGGTATTCACATGCCACAGG	323	54
3	rs31512068	**F:** TCGGGCATAGTCTCTGGTTC<br>**R:** GCCAGGGAACTACAACTCCA	445	58
4	rs13459075	**F:** TGAAGAGTCAGGCAGAAGCA<br>**R**: CTAAGGAGCAGACCCAGCAC	330	58
5	rs3664494	**F:** GAGCTTGGAAGGGGAAGG<br>**R:** AAATGAAGGGAGGCGACATA	330	54
6	rs13472545	**F:** CCAGCCCACAGTGAGTTGTA<br>**R:** GGGAAACCACAAAGACAGGA	492	58
7	rs8237423	**F:** GCCCTTCTGTCTCCCTCTTC<br>**R:** CCCAGACACTAGATCGCACA	492	58
8	rs13479805	**F:** ATAACCAGCTGAGGGTGTGC<br>**R:** ATGAGAGCCACATGGAGGAG	310	54
9	rs13480095	**F:** GACAGGCACAGCAAGGTACA<br>**R:** CCTGTGGATCTCACCTGTCA	377	58
10	rs13480662	**F:** CCCTGCTGTCTTTCTCTGCT<br>**R:** TGTGGGGGACATCTTCATCT	365	58
11	rs26891750	**F:** ATGAAGGCTGCAGGAAAAGA<br>**R:** CCAAAGAAGGACCCTGTTCA	346	54
12	rs29196570	**F:** ACACAGAACGGTCAGGTGGT<br>**R:** CGGAGAGGGGCATACATAAG	352	58
13	rs13481715	**F:** CCAGGAGTGTGTCTGCTCAA<br>**R:** GCAGAGTTGCCTGAGAATCC	423	58
14	rs30707092	**F:** GTTGCCACTTCTTCCTCTGC<br>**R:** CACATTTCGTGGGTCACAAG	412	58
15	rs13482486	**F:** ACTTAGTCATGGGCGGGTTT<br>**R:** TTGCCCTGACACTTGACATC	431	58
16	rs4163196	**F:** ACGGAGGTGTGTTCTGGTGT<br>**R:** GAGATGGGCAGAGGAGAGTG	377	58

17	rs3023450	**F:** CCATCCCTTTTATGCCTCCT<br>**R:** CACCTCCTTCTTGCTCACCT	349	58
18	rs29882799	**F:** ACCGGGAAGAACTGGAAACT<br>**R:** AGCCACACAGAGGAACAACA	300	58
19	rs4223757	**F:** TGCAAGACTGTCAGGAGGTG<br>**R:** CTGCCCACACTGGTTACCTT	352	58
X	rs13483822	**F:** CCGACTGTTCCCAAAACACT<br>**R:** GCATTTGCTACTGGGATGCT	382	54

Table S2. The results of SNP genotyping of parental HM-1 ES and cells (129sv strain) somatic cells (DBA2J strain) and fusion hybrid cells.

Chromosome number	SNP name	129/Ola (HM-1 ES cells)	DBA/2J (somatic cells)	Fusion hybrids	
				Clone 2.1	Clone 4.2
1	SNPa1	C	T	CT	CT
2	SNPb2	C	T	CT	CT
3	SNPa3	C	T	CT	CT
4	SNPa4	G	T	GT	GT
5	SNPb5	A	G	AG	AG
6	SNPa6	A	C	AC	AC
7	SNPa7	A	C	AC	AC
8	SNPb8	C	T	CT	CT
9	SNPa9	A	C	AC	AC
10	SNPa10	G	T	GT	GT
11	SNPa11	C	T	CT	CT
12	SNPa12	A	G	AG	AG
13	SNPa13	C	T	CT	CT
14	SNPa14	A	T	AT	AT
15	SNPa15	C	T	CT	CT
16	SNPa16	A	G	AG	AG
17	SNPa17	C	T	CT	CT
18	SNPa18	C	T	CT	CT
19	SNPa19	A	G	AG	AG

Table S3. PCR primers used for gene expression analyses.

Gene[a]	Primer	Sequence (5´to 3´)
GAPDH	Forward	GTGTTCCTACCCCCAATGTG
	Reverse	CTTGCTCAGTGTCCTTGCTG
Mlc2v	Forward	TGCCAAGAAGCGGATAGA
	Reverse	CAGTGACCCTTTGCCCTC
αMHC	Forward	ACCTGGGCAAGTCTAACAAC
	Reverse	CTGGATTCTGGTGATGATACG
cTnT	Forward	CTGAGACAGAGGAGGCCAAC
	Reverse	TTCTCGAAGTGAGCCTCGAT

[a] Abbreviations: GAPDH: glyceraldehyde-3-phosphate dehydrogenase; MLC2v: myosin light chain 2 ventricular; αMHC: alpha myosin heavy chain; cTnT: cardiac troponin T.
